# The Role of Beta-Adrenergic Receptors in Depression and Resilience

**DOI:** 10.3390/biomedicines10102378

**Published:** 2022-09-23

**Authors:** Hongxing Zhang, Mengqiao Cui, Jun-Li Cao, Ming-Hu Han

**Affiliations:** 1 Jiangsu Province Key Laboratory of Anesthesiology, Xuzhou Medical University, Xuzhou 221004, China; 2 Jiangsu Province Key Laboratory of Anesthesia and Analgesia Application Technology, Xuzhou Medical University, Xuzhou 221004, China; 3 NMPA Key Laboratory for Research and Evaluation of Narcotic and Psychotropic Drugs, Xuzhou Medical University, Xuzhou 221004, China; 4 Department of Mental Health and Public Health, Faculty of Life and Health Sciences, Institute of Brain Cognition and Brain Disease, Shenzhen Institute of Advanced Technology, Chinese Academy of Sciences, Shenzhen 518055, China; 5 Department of Pharmacological Sciences, Icahn School of Medicine at Mount Sinai, New York, NY 10029, USA

**Keywords:** beta-adrenergic receptors, major depressive disorder, stress resilience, antidepressant

## Abstract

Norepinephrine is a catecholamine neurotransmitter that has been extensively implicated in the neurobiology of major depressive disorder (MDD). An accumulating body of evidence indicates that investigations into the action of norepinephrine at the synaptic/receptor level hold high potential for a better understanding of MDD neuropathology and introduce possibilities for developing novel treatments for depression. In this review article, we discuss recent advances in depression neuropathology and the effects of antidepressant medications based on preclinical and clinical studies related to beta-adrenergic receptor subtypes. We also highlight a beta-3 adrenergic receptor-involved mechanism that promotes stress resilience, through which antidepressant efficacy is achieved in both rodent models for depression and patients with major depression—an alternative therapeutic strategy that is conceptually different from the typical therapeutic approach in which treatment efficacy is achieved by reversing pathological alterations rather than by enhancing a good mechanism such as natural resilience. Altogether, in this review, we systematically describe the role of beta-adrenergic receptors in depression and stress resilience and provide a new avenue for developing a conceptually innovative treatment for depression.

## 1. Introduction

Major depressive disorder (MDD) is a common psychiatric disorder that severely impairs an individual’s psychosocial functioning and the quality of daily life. It is known that the lifetime prevalence of MDD is roughly 20%, which means that one in five people experience an episode of depression at some point in their lifetime [1]. The World Health Organization (WHO) predicts that MDD will be ranked as the first global burden of disease by 2030 [1]. Regarding the treatment reality, less than half of all patients with MDD achieve remission with the currently available antidepressants [2]. Moreover, one-third of them fail to respond to conventional antidepressant medications and develop treatment resistance [3]. However, the development of new drugs to treat MDD has been unexpectedly slow for decades.

MDD is a mental illness of multiple etiologies involving genetic, epigenetic, environmental, psychosocial, and other risk factors [1]. Understanding the neuropathology of MDD has significantly progressed, but the translation of scientific findings from preclinical research to clinical practice has proved challenging. Currently available antidepressant agents for the treatment of MDD and other psychiatric disorders were serendipitously discovered in the mid-20th century, which led to the monoamine hypothesis of depression [1]. This theory has been supported by findings that antidepressant medications increase the levels of monoamine neurotransmitters (serotonin, noradrenaline, and dopamine) at synapses [4]. Accordingly, the theory also indicates an essential role of monoamine-releasing brain structures in the neuropathology and treatment of depression, including the midbrain dopamine system, the dorsal raphe serotonin system, and the locus coeruleus norepinephrine system. Interestingly, while the level of monoamines quickly increases once antidepressants are administrated, it usually takes weeks to months for them to exert clinical pathology-reversal effects [5]. This divergence indicates the sophisticated underpinnings of MDD and the more complicated mechanisms that may underlie the antidepressant effects of current medications.

At the beginning of the 21st century, ketamine, a noncompetitive N-methyl D-aspartate (NMDA) receptor antagonist and well-known general anesthetic, was identified as a rapid-onset antidepressant agent with persistent pharmacological effects in both animals and human beings with depression [6,7,8]. Additionally, esketamine, one of the enantiomers in the racemic mixture, was recently approved for treatment-resistant depression (TRD) by the US Food and Drug Administration [9]. Other compounds with similar properties, such as 3,4-methylenedioxymethamphetamine (MDMA) and psilocybin, have also attracted growing interest in preclinical studies and clinical investigations [9]. These compounds’ rapid onset and long-lasting antidepressant effects open a new time window for the treatment and mechanistic dissection of MDD. 

Interestingly, there are evident individual differences in response to adversity, trauma, tragedy, threats, or significant sources of stress: only a relatively small subgroup of the population develops psychiatric disorders (susceptibility), whereas the rest of them stay asymptomatically stable (resilience) [10]. Animal paradigms segregating susceptible and resilient subpopulations, such as the chronic social defeat stress (CSDS) model, have opened a novel avenue for the study of depression neurobiology and drug development for depression treatment [10,11,12,13,14,15]. For example, our recent work showed that resilience to repeated social stress was mediated by the mesolimbic dopamine neurons projecting from the ventral tegmental area (VTA) to the nucleus accumbens (NAc) [16] which leads to the successful translation of retigabine (an opener for the KCNQ subtype of K^+^ channels) from preclinical resilience mechanism studies to clinical depression treatment [12,13,14,17]. 

The results of early studies supported the idea that the disruption of the locus coeruleus–norepinephrine (LC–NE) system is strongly associated with psychiatric disorders, such as MDD and post-traumatic stress disorder (PTSD) in humans [18,19,20]. For example, rapid activation by various acute stressors is a highly conserved and adaptive function of the LC. These stressors, intrinsic and extrinsic to the individuals, activate the LC to release NE in its downstream targets, including the VTA, prefrontal cortex (PFC), amygdala, and hippocampus, which are related to psychiatric disorders [21]. Thus, persistent hyperactivity of the locus LC–NE system is believed to contribute to or be a risk factor for psychiatric disorders [22] and has been observed in patients with panic disorders, PTSD, and MDD [22,23,24]. Additionally, mice susceptible to a repeated traumatic experience with reminders of stress displayed an increased expression of the c-Fos protein in the LC. In contrast, resilient mice showed a similar c-Fos protein expression pattern relative to the control mice [22]. Recently, the LC–NE system was reported to be consistently and explicitly activated in mice resilient to chronic social stress, and the optogenetic mimicry of this hyperactivity was sufficient to generate resilience-like behaviors in behavioral measurements [25,26]. The divergence of alterations in LC noradrenergic activity is possibly due to the robust anatomical and functional heterogeneity of the LC–NE system, as well as the difference of animal models [27]. Together, the evidence described above indicates an emerging role of the norepinephrine–adrenoceptor system in resilience to depression beyond its already-known contributions to the neuropathology of depression itself.

Here, focusing on the beta-adrenoceptors, we reviewed the recent advances in depression neuropathology and the antidepressant effects related to different adrenoceptor subtypes. We also highlighted a beta-3 adrenoceptor that is involved in mediating stress resilience and has the potential to be a new drug target to promote a resilience mechanism and achieve antidepressant efficacy. We believe that investigations into the neurobiology of resilience to depression will pave a new avenue for developing conceptually novel therapeutic strategies for depression treatment.

## 2. Distribution and Biological Functions of Beta-Adrenergic Receptors

Adrenoceptors, a superfamily of guanosine triphosphate-binding protein (G protein)-coupled receptors (GPCRs), are receptors activated by epinephrine and norepinephrine [28]. According to its pharmacological properties, amino acid sequence, and signaling mechanisms, the adrenoceptor superfamily is classified into three subfamilies: the alpha1-, alpha2-, and beta-adrenoceptor subfamilies. The beta-adrenoceptor subfamily comprises the beta-1, beta-2, and beta-3 subtypes [28]. Beta-adrenoceptors are widely distributed in the nervous and non-nervous systems and are involved in the adaptation and maladaptation to social and environmental stimuli, as well as muscular system-related activities such as vasorelaxation and bronchodilation [25,28,29,30]. 

The beta-1 subtype of adrenoceptors is predominantly expressed in cardiac tissue and is critically involved in the regulation of the cardiovascular system in response to catecholamines [31,32,33]. In the central nervous system, beta-1 adrenoceptors are distributed in the basal lateral amygdala and cerebral cortex, playing a role in regulating symptoms of mental disorders, including depression and anxiety [34,35]. Beta-2 adrenoceptors are distributed throughout many areas, including smooth muscles, e.g., in the bronchi, veins, and gastrointestinal tract, to mediate smooth muscle relaxation-related physiological responses such as bronchodilation. They are also found in adipose tissue to regulate lipolysis and depressive-like symptoms [36,37]. Beta-3 receptors are thought to be mainly located in adipose tissue, where they are involved in enhancing lipolysis [38,39]. Some beta-3 receptor agonists, such as SR58611A and CL316,243, have been demonstrated to function in the regulation of behaviors related to neuropsychiatric disorders in animal studies [25,30], suggesting the expression and potential role of the beta-3 subtype receptors in the central nervous system. Beta-3 adrenoceptors in the gallbladder, urinary bladder, and skeletomuscular system control the relaxation of smooth muscle, and those in the skeletal muscle are used for thermogenesis [40,41,42]. The significant expression of beta-3 adrenoceptors has also been identified in cardiac tissue, indicating a role in the function of cardiovascular systems [43]. The distribution and functions of the three adrenoceptor subtypes are briefly summarized in Table 1.

## 3. Functional Role of Beta-Adrenergic Receptors in Depression and Antidepressant Effects

In the past few decades, clinical investigations have supported the expression and binding ability of beta-adrenoceptors in the leukocytes of patients with depression, supporting the role of peripheral beta-adrenoceptors in the prediction, development, and treatment effects of antidepressant therapeutics [49,50,51,52,53,54]. For instance, a lower density of beta-adrenoceptors in intact leukocyte cells in depressed patients is related to depression severity [55,56,57,58]. This lower density is also associated with a stable clinical improvement following antidepressant treatment [59]. A decrease in leukocyte beta-adrenergic receptor binding was also reported in depressed patients compared to healthy controls [60]. Inconsistently, a clinical trial performed on 20 depressed patients and 18 healthy volunteers measured the function of beta-adrenoceptors in lymphocytes, a subtype of leukocytes, with the radio-ligand binding technique. The results demonstrated the significantly up-regulated affinity and sensitivity of these receptors, which were decreased following electro-acupuncture-treatment in those showing strong therapeutic responses [61]. Furthermore, a beta-adrenoceptor binding study in lymphoblastoid cell lines from families affected by manic-depressive disorder demonstrated a 50% beta-adrenoceptor binding deficit compared to their unaffected relatives or healthy controls [62], indicating that beta-adrenoceptors are a potential biomarker in individuals who are genetically susceptible to these conditions.

The genetic variation of beta-1 adrenoceptors might influence clinical response to a specific antidepressant. A clinical study discerned the influence of the G1165C polymorphism in the beta-1 adrenoceptor gene on individual differences in response to sertraline, and it was found that CC genotype-carrying individuals responded five times more to sertraline compared to other variants and that the C allele carriers responded three times more to sertraline than patients with the G allele [63]. Interestingly, the antidepressant-promoting effect of polymorphism in the beta-1 adrenoceptor gene was not observed in patients who received repeated treatment with fluoxetine, one of the most frequently prescribed antidepressants [64]. Moreover, the antidepressant-induced down-regulation of CNS beta-1 adrenoceptors in rodents was in a time course that paralleled the onset of antidepressant action in patients with MDD [65]. The results of these studies demonstrate a causal link between beta-1 adrenoceptors and clinical antidepressant responses. 

Divergent data regarding the expression and role of beta-2 adrenoceptors have been reported in the leukocytes of depressed patients and after antidepressant treatment. For example, a clinical study indicated a significantly decreased beta-2 adrenoceptor density in unipolar patients compared to their controls, which demonstrated a significant association with the severity of the depression as assessed by the Hamilton Depression Rating Scale (HDRS) score [55]. The authors of another study failed to detect any change in leukocyte beta-2 adrenoceptors in untreated patients with depression. Still, they surprisingly observed increased beta-2 adrenoceptors following electroconvulsive therapy, indicating that the increased beta-2 adrenoceptors are a probable secondary effect of the lower circulating plasma noradrenaline [66,67]. The pharmacological activation of beta-adrenoceptors with salbutamol, a beta-2 adrenoceptor agonist, induced rapid antidepressant effects in patients with depression [68,69]. Animal studies have further demonstrated that the effect could be antagonized by beta-blockers [70], indicating a beta-2 adrenoceptor-dependent effect.

Compared to the subtypes described above, less is known about the role of beta-3 adrenoceptors in depression and the effects of currently available antidepressants on them. A recent rodent study showed that the systematic administration of SR58611A, a beta-3 adrenoceptor agonist that can penetrate blood–brain barrier, was able to significantly increase social interaction duration, reduce forced-swimming immobility, and effectively improve the degradation of the physical state of the animals’ coats following a long-term exposure to stress (33 days) [30]. All the behavioral data obtained in this study strongly indicated an antidepressant effect of the drug. Further studies of knockout mice suggested that these effects of SR58611A are mediated by beta-3 adrenoceptors [30]. These findings indicate that the pharmacological activation of beta-3 adrenoceptors with highly selective agonists [30,71] may represent an innovative approach for the treatment of anxiety and depressive disorders. 

Brain beta-adrenoceptor alterations have been suggested in suicide and MDD. For instance, beta-adrenergic binding studies performed in the frontal cortex and other brain samples from suicide victims have reported various changes compared to matched controls [72,73,74,75,76,77]. However, much less is known about the functional role of brain beta-adrenoceptors in these conditions, which needs to be addressed in future studies. 

Given their global expression in the cardiovascular, respiratory, and central nervous systems (Table 1), the systematic administration of beta adrenoceptor agonists or antagonists will evoke related side effects, such as cardiac disorders, trembling, nervous tension, headaches, and muscle cramps. In this case, for clinical translation, the side effects of beta adrenoceptor-related drugs should be carefully evaluated. Even administration through inhalation (which has advantages over oral or subcutaneous strategies) still leads to adverse effects such as bronchodilation [78,79]. Hyperthyroidism and diabetes mellitus are contraindications for the use of beta adrenoceptor agonists [80]. Drug interactions should also be taken in consideration, especially when administrated with corticosteroids, diuretics, and xanthine derivatives, because of the vibration of potassium levels [81]. In Table 2, Table 3, Table 4 and Table 5, we summarize currently-tested agonists and antagonists of beta-adrenoceptors in animal and human studies.

## 4. Functional Role of Beta-Adrenergic Receptors in Resilience to Stress

It is widely known that the LC–NE system globally primes neurons in the brain to respond to stimuli [94,95,96], e.g., in the fight or flight responses [97,98]. Emerging evidence suggests an essential role of the LC–NE system, consisting of norepinephrine and its adrenoceptors, in depression and stress resilience [25,26,94,95,96]. An accumulating body of evidence indicates a potential role of beta-adrenoceptors in mediating depression and the antidepressant effect of currently available treatments. However, the functional role of the LC–NE system in stress resilience is not well-known, and further investigations are required with etiologically and ethologically validated animal models. Here, we mainly presented the potential role of beta-3 adrenoceptor in mediating resilience to chronic social stress-induced depression. 

Individual variation in response to chronic social stress has been observed in a 10-day CSDS paradigm [17]. The CSDS model involves subjecting mice to repeated social defeat 5–10 min each day for 10 days, after which 60~70% of mice show a range of depressive-like behavioral symptoms (susceptible sub-population). In contrast, the rest of the socially defeated mice exhibit behavioral phenotypes similar to stress-naïve control mice (resilient sub-population) [12,16,17,25,99,100,101,102,103]. The segregation of the behaviorally and neurobiologically distinct sub-populations has led to an increasing body of basic and translational studies that have investigated the neural mechanisms of the resilience phenotype and provided the high potential to develop conceptually novel therapeutics achieved by promoting resilience mechanisms [10,11,12,13,14,15]. By utilizing the CSDS mouse model for depression, we and others recently found that LC norepinephrine neurons projecting to the VTA selectively exhibit enhanced neuronal activity in resilient mice [25,26]. Mimicking this firing adaptation by optogenetically activating these neurons in susceptible mice, 20 min per day for 10 days, reversed their depression-related behaviors and induced a homeostatic balance between *I*_h_ (hyperpolarization-activated cation channel current) and voltage-gated potassium (K^+^) currents in NAc-projecting VTA dopamine neurons. Our circuit- and cell-type-specific molecular profiling study further revealed that alpha-1 and beta-3 adrenoceptors displayed a significantly higher expression in VTA–NAc-projecting dopamine neurons. Subsequently, the pharmacologic activation of alpha-1 and beta-3 receptors in the VTA via the local infusion of a cocktail of their agonists was found to induce resilience phenotypes (methoxamine HCl and CL316243, once a day, for 10 days), an effect seen in optogenetically treated susceptible mice. Significantly, these optogenetic activation-induced pro-resilience effects were entirely blocked by the intra-VTA infusion of alpha-1 and beta-3 adrenergic receptor antagonists. These results strongly support the idea that alpha-1 and beta-3 adrenergic receptors are sufficient and necessary to mediate the resilience phenotype in the brain’s reward system. The repeated activation of VTA alpha-1 and beta-3 adrenoceptors displays pro-resilient effects in susceptible mice at the cellular and behavioral levels. For translational purposes, further studies are needed to independently examine the role of each receptor.

Together with the findings in non-social stress animal models [30], the evidence supports the idea that pharmacologically targeting the beta-3 adrenoceptors holds potential for developing novel antidepressants for depression treatment, which is achieved by promoting resilience-like phenotypes in chronic stress models of depression. 

## 5. Resilience Promotion as a Conceptually Novel Strategy for Depression Treatment

According to the American Psychological Association (APA), resilience is “the process of adapting well in the face of adversity, trauma, tragedy, threats, or even significant sources of stress”. An increasing body of evidence in animal studies and human investigations shows that resilience is an active, adaptive process rather than the absence of pathological responses, as seen in susceptible individuals. Several molecular and neurophysiological studies have consistently demonstrated that resilience actively recruits more genes and more ion channels to stabilize neuronal activity and maintain normal behavioral phenotypes [16,17].

The segregation of stress susceptibility versus resilience made it possible to identify multiple underlying neural and molecular correlates of resilience for the development of new treatments [10,11,12,13,14,15]. Focusing on the midbrain dopamine system, our recent preclinical and clinical studies have consistently supported the idea that resilience promotion is a bona fide strategy for conceptually novel drug development. NAc-projecting VTA dopamine neurons display increased firing activity in susceptible but not resilient mice. Optogenetically manipulating the neuronal activity of this subpopulation of VTA dopamine neurons was shown to bi-directionally regulate depressive-like behaviors in social interaction and sucrose preference tests [100]. Further mechanical investigations revealed that in susceptible mice, VTA–NAc dopamine neurons displayed an increased excitatory *I*_h_ current, which drives the pathological hyperactivity of these neurons [16]. Unexpectedly, in the resilient subpopulation, these neurons displayed an even larger excitatory *I*_h_ current that was accompanied by increased inhibitory K^+^ channel currents [16]. These findings indicate intrinsic homeostasis in the midbrain dopamine neurons involving an excitatory/inhibitory current re-balance that underlies resilience to repeated social stress. These findings also support the idea that targeting these K^+^ channels might exert an antidepressant effect by promoting resilience.

Our previous microarray study identified the KCNQ subtype of K^+^ channels as one of the four upregulated K^+^ channels selectively observed in the VTA of resilient mice [17]. Friedman et al. further demonstrated that bath application of the KCNQ opener retigabine (also known as ezogabine) suppressed the hyper-firing activity of VTA dopamine neurons in the brain-slice preparation obtained from susceptible mice. Moreover, both the viral overexpression of KCNQ3, a subunit that is upregulated in resilient mice, and the local infusion of retigabine into the VTA induced antidepressant properties in susceptible mice [12]. Further in vivo studies also demonstrated robust antidepressant effects in susceptible mice that received the systematic administration of retigabine, indicating a translational potential of this drug for clinical use [12]. Interestingly, targeting KCNQ4 with its openers was also reported to significantly decrease the hyperactivity of VTA dopamine neurons and abolish depressive-like behaviors in susceptible mice following CSDS [104].

Thus, an open-label clinical trial was performed to assess the effects of the KCNQ channel opener ezogabine on reward circuitry and clinical symptoms in 18 patients with MDD. After repeated exposure to ezogabine (up to 900 mg/day for 10 weeks), subjects exhibited significant reductions in depressive and anhedonia symptoms and a decreased functional connectivity between the ventral caudate and clusters within the mid-cingulate cortex and posterior cingulate cortex [13]; these findings highlight the KCNQ-type K^+^ channel as a promising target for future drug discovery efforts. In a later randomized placebo-controlled clinical trial involving 45 patients with depression, ezogabine treatment was associated with a robust improvement in depressive and hedonic symptoms compared to a placebo [14].

Together, these systematic findings identified KCNQ as a resilience-promotion target for conceptually novel antidepressants that function through the potentiation of active resilience mechanisms. 

## 6. Conclusions and Discussion

MDD affects many people, has an enormous impact on the individuals, imposes an immense economic burden, and suggests that the development of more effective treatments to improve its diagnosis and management is imperative. To achieve this goal, preclinical and clinical researchers have put forth enormous efforts over decades to identify pathophysiological alterations and reverse them to achieve therapeutic efficacy [5]. However, these strategies have not been successful and have failed to introduce new classes of antidepressants with novel mechanisms of action, and most currently available drugs for depression treatment were serendipitously discovered in the 1950s [9]. 

The segregation of susceptible versus resilient animals subjected to chronic stress is crucial, which provides two directions for future antidepressant development: to reverse the mechanisms specifically underlying susceptibility and to promote the active mechanisms underlying stress resilience. With the identification of ezogabine as a novel antidepressant that uses the resilience-promoting mechanism, the latter direction has been proven to be a successful approach for the development of possible new-generation antidepressants, which clearly is a conceptually novel therapeutic strategy for MDD. An accumulating body of evidence from preclinical and clinical investigations strongly supports the idea that beta-adrenoceptors, especially the beta-3 subtype, hold promising possibilities as molecular targets for future antidepressant development. Therefore, agonists that are highly selective for beta-3 adrenoceptors may represent druggable compounds for future preclinical and clinical tests [30,71]. Based on our current knowledge about resilience, targeting resilience-related channels or receptors in the brain represents an evidently different mechanism of action compared to any currently available antidepressants. Hopefully, more brain structures and molecules underlying resilience to chronic stress will be identified, which will offer conceptually original therapeutic strategies for depression treatment.

## Figures and Tables

**Table 1:** A summary of the distribution and functions of the three beta-adrenoceptor subtypes.

Beta-Adrenoceptor Subtypes	Distribution	Function/Effect	Reference
Beta-1	Cardiac tissue	Heart rate, heart failure, hypotension	[31,32,33]
	The central nervous system, e.g., the basal lateral amygdala and the cerebral cortex	Psychiatric disorders, e.g., anxiety	[34,35]
	Adipose tissue	Lipolysis	[31]
	Salivary glands	Amylase release	[44]
Beta-2	Smooth muscles e.g., in the bronchi, the veins, and the bladder	Smooth muscle relaxation-related effects	[43,45,46]
	Adipose tissue	Lipolysis and mental disorders, such as depression	[36,37]
	Pancreas	Insulin and glucagon secretion	[47]
Beta-3	Adipose tissue	Lipolysis	[38,39]
	Smooth muscles e.g., in the bronchi, the veins, bladder, and the gastrointestinal tract	Smooth muscle relaxation-related effects	[40]
	Cardiac tissue	Heart failure and hypertension	[43]
	The central nervous system, e.g., the ventral tegmental area and the prefrontal cortex	Psychiatric disorders, e.g., depression	[25,48]
	Skeletal muscle	Thermogenesis	[40,41,42]

**Table 2:** Beta-adrenoceptor agonists tested in animal studies.

Agonists	Receptor Target	Administration Strategies	Behavioral Tests	Function	Animal Species	Reference
Methoxamine and CL316243 cocktail	Alpha-1 and Beta-3	0.02 μg and 0.6 μg, VTA infusion, 10 days	SIT	Antidepressant	mouse	Zhang et al. [25]
SR58611A	Beta-3	1/3/10 mg/kg, i.p., 10 days	FST, SIT	Antidepressant	mouse/rat	Stemmelin et al. [30]
SR58611A	Beta-3	5/10 mg/kg, i.p., 24/5/1 h prior to behavioral tests	FST, SIT	Antidepressant	rat	Consoli et al. [82]
SR58611A	Beta-3	0.3/1.0/3.0 mg/kg, i.p., 14 days	FST	Antidepressant	rat	Overstreet et al. [83]

SIT, Social Interaction Test; FST, Forced Swim Test; i.p., intraperitoneal. SIT and FST are well-established behavioral tests used to evaluate depressive-like symptoms related to behavioral despair and social avoidance. Another widely-used test is the sucrose preference test, which measures the change of anhedonia in tested animals.

**Table 3:** Beta-adrenoceptor antagonists tested in animal studies.

Antagonist	Receptor Target	Administration Strategies	Behavioral Tests	Function	Animal Species	Reference
Nebivolol	Beta-1	10 mg/kg p.o., daily	OFT, FST	Antidepressant	rat	Abdelkader et al. [84]
Cyclazosin and SR59230A	Alpha-1 and Beta-3	0.2 μg and 0.02 μg, VTA infusion, 10 days	SIT	Pro-depression	mouse	Zhang et al. [25]
SR59230A	Beta-3	5 mg/kg i.p., twice daily for 10 days	SIT	Pro-depression	mouse	Chuang et al. [85]

OFT, Open Field Test; FST, Forced Swim Test; SIT, Social Interaction Test.

**Table 4:** Beta-adrenoceptor agonists tested in human studies.

Agonists	Receptor Target	Administration Strategies	Clinical Symptom Evaluation	Patients	Reference
Salbutamol	Beta-2	1.5~6 mg intravenous infusion, 6~10 days	HDRS	MDD	Simon et al. and Lecrubier et al. [68,70]
SR58611A	Beta-3	350 mg q12, 12 weeks	CGI-S, MADRS, HAM-A	MDD	Sanofi-NCT00252330 *

HDRS, Hamilton Depression Rating Scale; CGI-S, the Clinical Global Impression-Severity scale; MADRS, the Montgomery–Åsberg Depression Rating Scale; HAM-A, Hamilton Anxiety Rating Scale; MDD, Major Depressive Disorder; * ClinicalTrials.gov Identifier (phase 3).

**Table 5:** Beta-adrenoceptor antagonists tested in human studies and clinical observations.

Antagonists	Receptor Target	Administration Strategies	Clinical Symptom Evaluation	Patients	Reference
Propranolol	Non-selective	45~120 mg daily, 2 weeks~3 months	Pro-depression *	Hypertension; angina- pectoris; other beta-blocker users	Waal et al., Oppenheim et al. and Thiessen et al. [86,87,88]
Timolol	Non-selective	0.25% twice a day, 3 months	Pro-depression *	Adult-onset diabetes and diabetic retinopathy	Nolan et al. [89]
Sotalol	Non-selective	80 mg twice daily, 4 months	Pro-depression *	ICD user	Ramaswamy et al. [90]
Betaxolol	Beta-1	One drop in each eye every 12 h	Pro-depression *	Glaucoma	Orlando et al. [91]
Metoprolol	Beta-1	23.75 or 47.5 mg, qd PO, dose escalated with 23.75 mg each time until target heart rate < 70 bpm was achieved	HADS, CBI	Chronic heart failure	Liu et al. [92]
Metoprolol	Beta-1	50/25/12.5 mg twice a day	Pro-depression *	Hypertension, hyperlipidemia, benign prostate hyperplasia, Barrett’s esophagus, mild dementia, and chronic back pain	Shah et al. [93]

HADS, Hospital Anxiety and Depression Scale; CBI, Copenhagen Burnout Inventory; ICD, implantable cardioverter-defibrillator; * Case report.

## Data Availability

Not applicable.
